# Biomechanics and ontogeny of gliding in wingless stick insect nymphs (*Extatosoma tiaratum*)

**DOI:** 10.1242/jeb.247805

**Published:** 2024-12-16

**Authors:** Yu Zeng, Grisanu Naing, Vivian Lu, Yuexiang Chen, Robert Dudley

**Affiliations:** ^1^ Department of Integrative Biology, University of California, Berkeley, Berkeley, CA 94720, USA; ^2^ Department of Integrative Biology, University of South Florida, Tampa, FL 33620, USA; ^3^ Department of Earth and Planetary Science, University of California, Berkeley, Berkeley, CA 94720, USA; ^4^ Smithsonian Tropical Research Institute, Balboa, Republic of Panama

**Keywords:** Aerodynamics, Arthropod, Body size, Forest canopy, Ontogeny, Legs

## Abstract

Many wingless arboreal arthropods can glide back to tree trunks following free falls. However, little is known about the behaviors and aerodynamics underlying such aerial performance, and how this may be influenced by body size. Here, we studied gliding performance by nymphs of the stick insect *Extatosoma tiaratum*, focusing on the dynamics of J-shaped trajectories and how gliding capability changes during ontogeny. After being dropped 40 cm horizontally from a visual target, the first-instar nymphs landed on the target within 1.1 s. After reaching terminal speed (at ∼0.25 s), they initiated gliding with significant horizontal force, during which the overall lift-to-drag ratio increased from 0.16 to 0.48. This transition from parachuting to gliding is characterized by a damped oscillation in body pitch, initiated with a rapid nose-down pitching, and led to a higher-lift configuration with reduced body angle of attack. Among instars, increasing wing loading during ontogeny led to greater terminal speed, reduced agility during glide initiation and increased glide angle. Our study demonstrates that a sequence of controlled behaviors, from pre-glide descent to glide initiation and forward gliding, underlies their gliding aerodynamics, which in aggregate form the basis for J-shaped aerial trajectories. Selection for improved gliding performance in wingless arthropods may foster the evolution of more rapid maneuvers and of dedicated morphological traits (such as winglets) that contribute to an overall reduction in wing loading, either across ontogeny or during the evolution of larger body size.

## Teaser

**Summary:** In under half a second, stick insect hatchlings swiftly adjust their leg posture, initiating controlled gliding from a fall.

## INTRODUCTION

Gliding is adopted by many wingless arboreal animals for accessing resources, escaping from threats, and dispersing within forest canopies (Dudley et al., 2007; Khandelwhal et al., 2023). Gliding capability has evolved many times in wingless arthropods, such as jumping bristletails, ant workers, and various wingless nymphs of winged insects and spiders (Yanoviak et al., 2009, 2011, 2015; Socha et al., 2015; Zeng et al., 2015; Zhao et al., 2023). Gliding and controlled manoeuvres may also have been an evolutionary precursor to powered flapping flight in insects (Dudley and Yanoviak, 2011). Whereas various wingless taxa are capable of gliding, force dynamics along glide trajectories and how they may be influenced by morphological traits (such as body size and body–leg shape) remain unclear.

Field experiments have revealed robust gliding performance in numerous wingless arthropods. For example, gliding ants dropped 1–2.5 m horizontally from a tree trunk can land on the tree trunk with an average glide index (i.e. horizontal distance traveled per unit vertical descent) ranging from 0.1 to 0.5 (Yanoviak et al., 2005, 2008). Glides in wingless arthropods are typically initiated following a free fall initiated via either startle jumping or self-dropping (Yanoviak et al., 2005, 2009; Humphreys and Ruxton, 2019; Zeng et al., 2020), and are typically characterized by J-shaped trajectories. These trajectories may be roughly divided into a transient and accelerating initial descent followed by an equilibrium gliding phase. This seemingly simple trajectory derives, however, from complex behavioral control and a transition from unsteady to steady-state aerodynamics.

First, pre-gliding transient descent is a phase during which the insect can perform rapid maneuvers and changes in body–leg postural configuration. Detailed kinematics of this initial stage are not known for most taxa. Nymphal stick insects dropped upside-down can perform rapid righting and subsequent post-righting stabilization within 0.3 s (Zeng et al., 2017). Once righted, the falling insect performs visually guided steering and glides toward a target at variable turning radii (Yanoviak et al., 2005, 2011). These directional maneuvers depend on recognizing suitable landing targets in their native habitat (Yanoviak and Dudley, 2006; Zeng et al., 2015). Although the detailed kinematics remain unclear, these maneuvers seem to occur early in gliding, presumably after the landing target is identified.

Second, the aerodynamics of gliding in wingless arthropods can be very different from that in larger animals, given that their body and leg segments consist only of rigid and approximately cylindrical structures with limited capacity for lift generation (Ellington, 1991). This constraint limits maximum horizontal force production, resulting in steep glide angles greater than 45 deg, as compared with relatively shallow glides in gliding vertebrates with well-defined aerodynamic surfaces (e.g. flying lizards and mammalian gliders; Socha et al., 2015). For this reason, wingless arthropods in gliding modulate their intrinsic stability within a largely upward flow field, for which a positive static stability is essential to falling through air in the presence of wind disturbance (see McCay, 2003). All of their aerial maneuvers thus derive from specific body–flow configurations at high body angles of attack (>45 deg), which is distinct from typical wings that best perform (e.g. reaching the maximum lift-to-drag ratio) at lower angles of attack (e.g. <40 deg), as also characterizes wing-based aerial vehicles (Nelson, 1998).

**Fig. 1.**
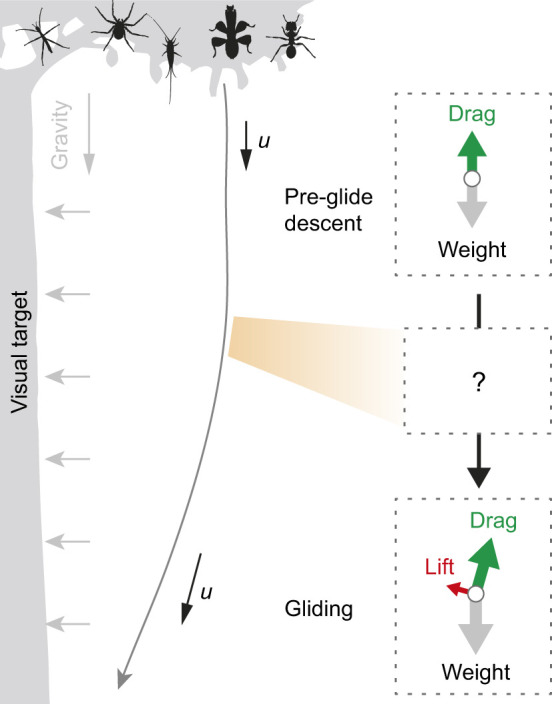
**Gliding mechanism and glide initiation in wingless gliding arthropods.** Different wingless gliding arthropods can initiate gliding during vertical descent, as mediated by the visual information associated with potential landing targets. Such trajectories are typically J-shaped, and the inflection of a J-shaped trajectory corresponds with a transition between two statically stable states with different aerodynamic configurations (see insets, with arrows showing forces on the center of mass; *u*, velocity). This study aims to address the behavioral and aerodynamic mechanisms underlying such J-shaped glide trajectories based on controlled laboratory experiments.

All wingless gliding arthropods initiate trajectory control during descent. Such a transition corresponds to the inflection of typically J-shaped trajectories, and can be seen from a lateral view as the falling arthropod gains horizontal speed and glides toward a target (Yanoviak et al., 2005, 2015; Zhao et al., 2023). As depicted in Fig. 1, glide initiation requires a transition from predominately vertical force dynamics (i.e. drag versus body weight) to a different configuration with sustained lift production. Experimental observations of parachuting behavior in both non-gliding ants and gliding nymphal stick insects show their ability to maintain a stable parachuting posture (Yanoviak et al., 2011; Zeng et al., 2017), but how a parachuting insect transitions to stable gliding remains unclear. To initiate gliding from parachuting, the insect should adjust its body–leg postural configuration to induce changes in aerodynamic characteristics (such as moment derivatives) and in the body's angle of attack. A potential analogy may be found in gliding parafoils, which can switch between different glide angles by changing the angle of attack (Slegers et al., 2008; Ward et al., 2013). Therefore, documenting body kinematics throughout the process of glide initiation can help to elucidate mechanisms of transition into equilibrium gliding.

Another underexplored phenomenon among wingless gliding arthropods is the reduction of gliding capacity with increased body size. Specifically, larger individuals land at lower heights than do smaller individuals of the same species (Yanoviak et al., 2005, 2008, 2009, 2015; Zhao et al., 2023). This outcome presumably derives from increased vertical speeds and terminal velocity in larger individuals, but how the dynamics of J-shaped trajectories are influenced by increased body size and also wing loading (i.e. the ratio of body weight to projected aerodynamic surface area) requires more detailed kinematic sampling. Ideally, one can sample gliding trajectories from the same species across different nymphal stages.

How wingless arthropods glide after initial free fall also demands detailed observations under controlled conditions. Such experiments can help to reveal how variable initial conditions (e.g. initial body orientation and velocities) and pre-glide behaviors may influence the dynamics of subsequent glide trajectories. In addition, although free-drop experiments have been used in testing the function of winglets on physical models of protopterygotes (i.e. the ancestors of winged insects; Wootton and Ellington, 1991), further experimental results from living insects will provide important comparative results for understanding the biomechanics of gliding with winglets, and of incipient flapping flight.

**Fig. 2.**
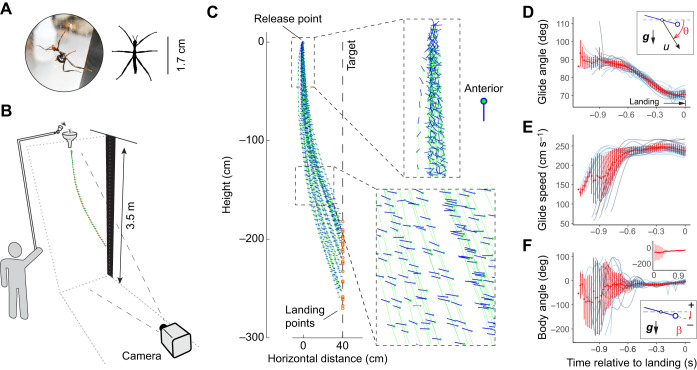
**Experimental setup and gliding performance in newly hatched *Extatosoma tiaratum* nymphs.** (A) Experimental arena. Insects were dropped through a plastic funnel positioned 3.5 m above the ground. All vertical walls were covered by white felt, and a narrow black strip (15 cm wide) was used as a visual target. In addition to the configuration shown here, other cameras were set up to capture close-ups of different phases of the glides. Inset, a newly hatched nymph. (B) Sample trajectories from trials of 0-DAH (days after hatch) nymphs (*N*=5 individuals; 5–8 trials per individual), illustrating pre-glide descent and the subsequent gliding phase. Trajectories of the center of body mass (green) were reconstructed based on positions of head and abdomen (see ‘Kinematics’ in Materials and Methods). Insets demonstrate the highly variable body orientations during the pre-glide descent, with a more consistent body orientation during near-equilibrium gliding (see also Movie 1). (C–E) Variation in glide angle (θ), glide speed (*u*) and body angle (β) for 0-DAH nymphs as plotted up to the point of landing, and showing convergence in body orientation and body–flow configuration toward the end of the glide. Red dots and error bars represent means and s.d., respectively, with individual trials illustrated in the background.

Here, we examined gliding performance by nymphs of the Australian stick insect, *Extatosoma tiaratum*, under controlled laboratory settings. Newly hatched *E. tiaratum* disperse from the forest floor to the canopy in the daytime (Fig. 2A), during which gliding behavior may facilitate gap crossing, and return to vegetation after falling initiated by perturbations (Zeng et al., 2020). When gliding, the nymphs preferentially target vertically oriented black stripes against a white background (Zeng et al., 2015), which we also used in this study as potential landing targets. Within an environmentally controlled enclosure, we filmed falls and gliding in *E. tiaratum* nymphs when dropped from a constant horizontal distance to the visual target. We evaluated aerial performance in four age groups within the first instar, so as to demonstrate ontogenetic variations in underlying behavioral and aerodynamic mechanisms. Also, from the first to the last instar stage, an *E. tiaratum* undergoes a 100-fold increase in body mass and 50-fold increase in wing loading (see also Fig. S1). We thus also evaluated how gliding ability varies across this full instar range.

## MATERIALS AND METHODS

### Insects and morphometrics

We used individuals from a laboratory colony of *Extatosoma tiaratum* (Macleay 1826) for experiments (see Zeng et al., 2020 for details). For first instar nymphs, we used four age groups: 0-DAH (i.e. the day after hatching), 5-DAH, 9-DAH and 15-DAH. For older nymphs, we used males and females in the second, fourth and sixth instars (4–5 individuals per instar per sex) on the sixth or seventh day following the prior molt.

Insect mass (*m*) was measured using an electronic balance (R200D, Sartorius AG, Germany). Digital photos were taken with a camera (E3 Olympus, Tokyo, Japan) for nymphs anesthetized with a 5-min CO_2_ treatment, and then positioned on a horizontal foam board with all legs laterally extended. Next, body length (*L*) and dorsally projected planform area (*A*) were measured from photos using ImageJ (Rueden et al., 2017). Following the conventional use of the term ‘wing loading’ (i.e. the ratio of weight to the area of aerodynamic surface) for gliding animals (see Dudley et al., 2007; Socha et al., 2015), we calculated wing loading of the body–leg system as *p*_b_=(*m**g***)/*A*, where ***g*** is gravitational acceleration.

### Experimental protocol

Filming of gliding in first instar nymphs was conducted within a temperature-controlled enclosure (24–27°C) at the Animal Flight Laboratory at UC Berkeley. Both the target wall (4 m high and 2 m wide) and a perpendicular background wall (4 m tall and 2 m wide) were covered by white felt. A 15 cm wide black felt stripe, which served as the landing target, was attached to the target wall (see Zeng et al., 2015 for reflectance profiles). A funnel was made from the top portion of a plastic bottle, with the inner wall coated with Teflon (no. 2871, BioQuip Products, Gardena, CA, USA). The funnel was suspended such that its nozzle was 3.5 m above the ground and 40 cm horizontally distant from the black felt stripe. Lighting was provided by halogen lamps. In experiments, 1st instar nymphs were individually dropped from a Teflon-coated cup into the funnel by the experimenter (Fig. 2B). Glide trials were filmed in lateral view using a high-speed video camera (500 Hz; HiSpec, FasTec Imaging, CA, USA; ∼3 pixels cm^−1^) set 1.7 m above the ground, and facing the background wall (Movie 1). In addition, we filmed numerous trials for preliminary observations of leg posture during different stages of gliding (in either top, oblique or lateral views) using high-speed cameras (Troubleshooter or HiSpec, FasTec Imaging).

### Kinematics

For insects filmed in lateral view while gliding, we tracked the anterior and posterior tips of the insect (i.e. head and distal segment of the abdomen) at 50 Hz by sampling every 10 frames, using commercial software (ProAnalyst, Xcitex Inc., MA, USA). For sequences exhibiting substantial pre-glide maneuvers, we tracked the landmarks in reversed frame sequence, so that the head and abdomen could be inferred from the gliding phase.

Kinematic analysis was conducted using custom-written scripts in MATLAB (R2019b, MathWorks, Natick, MA, USA) and R (https://www.r-project.org/). Raw trajectory data were first processed with quintic spline (MATLAB's *spaps* routine; tolerance *tol*=0.5), which is a two-dimensional analog of the optimal method (‘MSE’ method; Walker, 1998). When presenting the general trend for different age groups, temporal data sequences were presented as assemblages of means with standard errors of measurement (i.e. s.e.m.), or as LOESS (locally weighted scatterplot smoothing) regressions (in R package ‘ggplot2’, with ‘span=0.5’; see Wickham, 2009). The center of mass (CoM) was approximated as the midpoint of the body length (see Zeng et al., 2017), and the body velocity (*u*) and glide angle (θ) were then calculated based on CoM translation (Fig. 2C). Based on the lateral projection of the longitudinal body axis (i.e. a vector connecting the head to abdominal tip), the body pitch angle (β) was calculated with respect to the horizontal (Fig. 2D); the body angle of attack (AoA) was calculated with respect to the direction of incident air flow.

Two temporal landmarks were used to segregate complete gliding behaviors (i.e. over the interval from dropping to landing) into different phases. First, we identified the maximum vertical speed (*u_z_*_,max_), and used a value of 95% *u_z_*_,max_ as the indicator for having attained terminal speed (Fig. S2). Recognizing that no sustained vertical force equilibrium was attained, we use the term ‘terminal speed’ to represent a state with relatively small changes in vertical acceleration during the late stage of gliding. Second, initiation of gliding was characterized by a large change in horizontal force (see below), which we identified as the moment of maximum horizontal acceleration (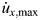).

To compare dynamics among insects of different masses, we used acceleration as a proxy for total force. We calculated accelerations using time derivatives of displacement data. The total force acting on the CoM is the sum of aerodynamic forces and gravitational force. The resultant total acceleration (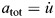) can be expressed as the vector sum of the corresponding accelerations (*a*_air_ and ***g***, respectively):
(1)

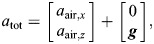
from which we calculated *a*_air_. The drag acceleration was calculated as the projection of the acceleration vector (*a*_air_) onto a unit vector (*n*_D_):

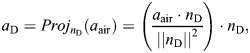

(2)

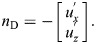
Similarly, lift acceleration was calculated as 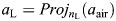, with 
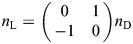
 being the unit vector of lift. For the damped oscillation of body pitch angle during glide initiation, we identified local peaks using the ‘find_peaks’ function in R (pracma; https://CRAN.R-project.org/package=pracma), and calculated a damping ratio using the logarithmic decrement method. Natural frequencies were found by performing fast Fourier transforms for body pitch angle, and then identifying the peak frequencies.

## RESULTS

### Gliding performance

After falling through the funnel nozzle, first instar nymphs quickly glided toward the visual target. Using the youngest 0-DAH nymphs as an example, initial body orientation was highly variable but quickly converged to an equilibrium and gliding-specific posture (–3.0±7.5 deg relative to horizontal; mean±s.d.), with a stereotypic glide speed (237.6±13.0 cm s^−1^) and glide angle (70.8±2.2 deg) (Fig. 2D–F). Laterally projected trajectories were generally J-shaped but varied in pre-glide height loss and in landing heights, even within the same individual (see below).

**Fig. 3.**
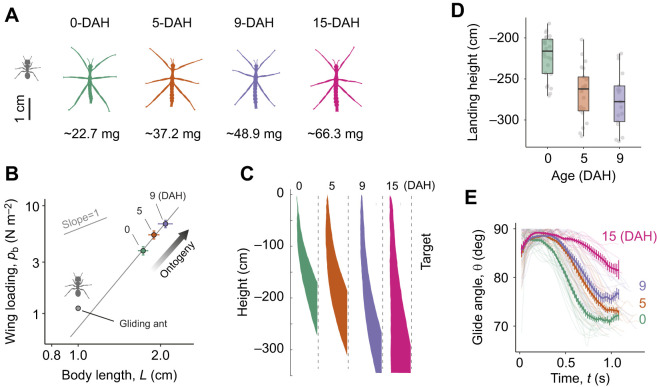
**Overall gliding performance in first instar nymphs of *E. tiaratum*.** (A) Dorsally projected planforms of first instar nymphs in four different ages, as compared with a gliding ant (worker of *Cephalotes atratus*, ∼1 cm in body length), with values below for mean mass (s.e.m. 0.42–0.85 mg). (B) Allometry of wing loading within the first instar. Values represent means±s.d. A mean power-law exponent of 1.8±0.4 is greater than expected from an isometric model (i.e. slope=1), resulting from an 20% increase in body length and ∼200% increase in mass comparing newly hatched with 9-day-old nymphs. Wing loading of *C. atratus* is based on Socha et al. (2015). (C,D) Aggregated trajectory shapes (representing all trajectories from the same age group) and height of landing points for four age groups, showing a downward shift as age increases (0-DAH, −222±27 cm; 5-DAH, −276±32 cm; 9-DAH, −298±57 cm; the 15-DAH group was excluded owing to ground impact in a large number of trials). (E) Temporal variation in glide angle showing an ontogenetic shift with instar age. In the 0-DAH group (green), a directional bias was evident ∼0.2 s after dropping of the insects. Trend lines are means with error bars representing s.e.m.; data from individual trials were plotted in the background.

As shown in Fig. 3A,B, the first instars nymphs underwent a three-fold increase of body mass (from 22.7 to 66.2 mg) with time but showed only a minor increase in projected area (from 66.1 to 72.1 mm^2^), resulting in a nearly two-fold increase of wing loading. With such increased wing loading, the aerial trajectory became more stretched vertically, with the height of landing points shifting downward. All 0-DAH (*N*=24 trials) and 5-DAH groups (*N*=21 trials) landed on the target; in the 10-DAH group, insects in 17 out of 21 trials landed on the floor; in the 15-DAH group, insects in all trials landed on the floor. The glide angle before landing increased from ∼71 to ∼82 deg (0-DAH, 70.6±1.9 deg; 5-DAH, 72.0±2.0 deg; 10-DAH, 73.7±3.2 deg; 15-DAH, 82.0±3.2 deg) (Fig. 3C–E). We first describe the general trajectory based on data for the 0-DAH nymphs, and then summarize patterns of ontogenetic variation.

### Main phases of the J-shaped trajectory

**Fig. 4.**
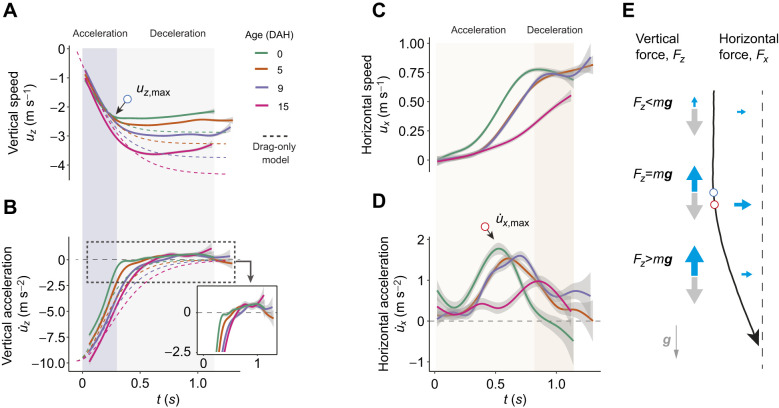
**Phases of body translation in gliding *E. tiaratum* nymphs.** Different aerial phases for differently aged nymphal groups. In the vertical direction, insects exhibit an initial downward acceleration (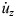
<0 m s^−2^) within the first 0.25 s after release, and subsequently experience a deceleration phase, as shown in A and B. The accelerations, as derived from both lift and drag (see Fig. 8), are distinct from those on a drag-only model (represented by dashed lines), as derived from aerodynamic forces acting on horizontal cylinders with the same mass and projected planform area as the insects (see Supplementary Materials and Methods, section A). (C,D) In the horizontal direction, insects showed initially low acceleration, followed by a rapid acceleration starting at ∼0.25 s and then leading to glide initiation. Curves represent LOESS regressions, with shading indicating s.e.m. Ontogenetic variation correlates with a greater terminal speed (A), reduced vertical acceleration (B) and delayed initiation of gliding (C,D). (E) A schematic summary of body dynamics in vertical and horizontal directions along a J-shaped trajectory. See also Fig. S2.

The vertical fall of the CoM is composed of two phases: (1) an initial downward acceleration and (2) a subsequent deceleration (Fig. 4A,B), with the transition when vertical speed reached a maximum (*u_z_*_,max_) at ∼0.25 s from the start of the fall. This velocity profile was distinct from that of a reference physical model with only drag (i.e. cylinders with approximately the same projected area and mass as the insects) (dashed lines in Fig. 4A), given that lift generation in actual insects provided substantial upward force (see below). In the horizontal direction, the speed of the CoM increased from ∼0.13 to ∼0.75 m s^−1^ over the time interval of 0.25–0.7 s, as driven by an increase in force peaking at ∼0.5 s (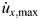) (Fig. 4C,D). This peak corresponded to the inflection of the J-shaped trajectories where pre-glide descent transitioned to gliding.

To analyze variance in trajectory shape and dynamics, we identified two prominently recognizable kinematic landmarks from all trajectories. First, we used the time to reach 95% terminal speed (i.e. 95% *u_z_*_,max_) as the start of near-equilibrium parachuting (blue circle in Fig. 4E). Second, we used the time of peak horizontal acceleration (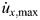) to represent the initiation of gliding (red circle in Fig. 4E; see also Fig. S2).

### Pre-glide maneuvers

**Fig. 5.**
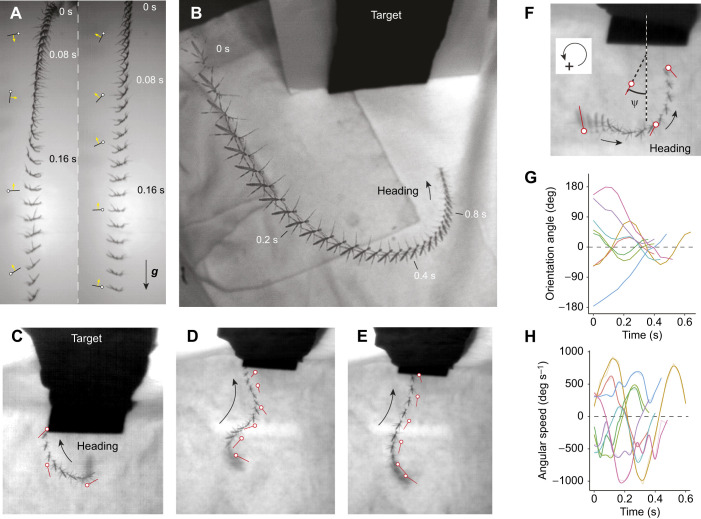
**Mid-air maneuvers by *E. tiaratum* nymphs.** (A) Two sample sequences of first instar nymphs performing aerial righting with different initial body orientations. Insects were dropped from Teflon-coated cups. (B) Sequence of steering toward the visual target prior to gliding, as filmed in oblique view. (C) Sample sequences of steering and maneuvering via yaw rotations prior to (left) and during gliding from mid- (D) and right (E) views. Red arrows denote body orientation; time increment is 40 ms. (F) Schematic for tracking of body orientation based on the head and abdominal tip, as demonstrated with a sample sequence as captured by a vertically oriented camera. The vertically projected body orientation angle (ѱ) was calculated as the angle between the longitudinal body axis and a vector normal to the plane of the visual target. (G) Vertically projected body orientation angle through time (*N*=8 trials). Note that the insect's body axis became more aligned with the target toward the end of the sequence. (H) Temporal variation in rotation speed of body axis (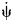) during steering. See also Movies 3, 4.

After falling through the funnel in a random body orientation, the insects performed two types of maneuvers to initiate forward gliding: (1) aerial righting to attain dorsoventral orientation and (2) reorientational steering to align orientations of the body axis and heading. In a previous study, 0-DAH nymphs dropped upside-down with no initial perturbation (i.e. with zero linear and angular momenta) completed dorsoventral righting within 0.2 s over a ∼30 cm loss in height, and with an average rotational speed of ∼900 deg s^−1^ (Zeng et al., 2017). Here, 0-DAH nymphs released from a flipped Teflon-coated cup showed faster initial body rotations and completed righting earlier, within 0.15 s (Fig. 5A; Movie 2).

Once righted in the air, the descending insect steered toward the visual target. The simplest steering was a simple turning movement in a circular path (Fig. 5B,C; Movie 3). In addition, we also observed mid-glide oscillation of the longitudinal body axis while the insect moved toward the target (Fig. 5D,E). Based on sampling of the angle between the longitudinal body axis and a vector normal to the target wall, the angular speed of such steering reached a maximum of 1000 deg s^−1^, with an average of 300–400 deg s^−1^ (Fig. 5F–H). These angular speeds indicate that 0.45–0.6 s is required to finish a 180 deg turn, corresponding to a height loss of 0.9–1.2 m (assuming a vertical speed of 2 m s^−1^). These rotational speeds during steering maneuvers are comparable to those of gliding ants (187–714 deg s^−1^; Yanoviak et al., 2010).

### Spatiotemporal variation in glide initiation

**Fig. 6.**
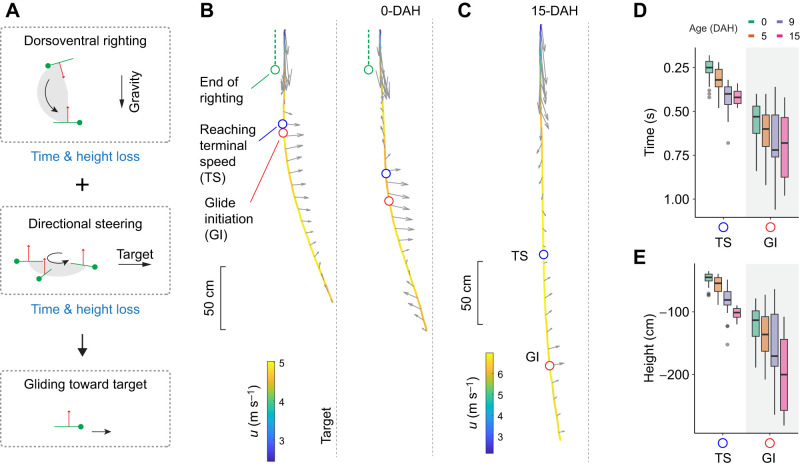
**Spatiotemporal variation in gliding initiation.** (A) The insects perform two maneuvers before gliding toward the target: (1) dorsoventral righting such that the dorsal direction (red arrow) is aligned upward, and (2) reorientational steering so that the body longitudinal axis (green line) is aligned with the line-of-sight to the target. After being dropped in haphazard orientations, and at greater angular distances from a forward gliding configuration, insects spend more time to complete maneuvers, and hence fall over greater distances. See also Supplementary Materials and Methods, section B and Fig. S3. (B) Two trajectories from the same individual at 0-DAH, but with different elapsed time and height loss when reaching terminal speed (TS) and glide initiation (GI). The maximum height loss at the end of righting is ∼30 cm. (C) The two kinematic landmarks in B also shifted downward through ontogeny, as demonstrated by one trajectory from an older individual (15-DAH). (D) Time and (E) height loss to reach TS and GI as summarized for differently aged insects, showing variation both within each age group and across ontogeny.

As depicted in Fig. 6A, depending on the initial body orientation relative to gravity and to the target, the insect may perform various combinations of dorsoventral righting and directional steering to achieve a head-first orientation facing the target. The time required for the insect to achieve the desired orientation is directly influenced by the extent of its initial deviation from the target direction. The greater the deviation in both the dorsoventral axis (relative to vertical) and the heading direction (relative to the line-of-sight to target), the longer it will take for the insect to align its longitudinal body axis toward the target. Although the kinematic data lacked three-dimensional resolution, our observations support the conclusion that variable initial conditions caused the variation in timing and heights at which gliding was initiated, even within the same individual (Fig. 6B; Fig. S3, Supplementary Materials and Methods, section B).

Based on the two kinematic landmarks, i.e. reaching terminal speed (95% *u_z_*_,max_) and the initiation of gliding (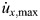) (Fig. S2), we found that the initiation of gliding phase always followed the acquisition of terminal speed. However, there was a scattered distribution for both landmarks (Fig. 6D,E). In the 0-DAH group, terminal speed was reached between 0.2 and 0.4 s and with a height loss of 50–70 cm; gliding initiation occurred between 0.4 and 0.85 s, with a height loss of 0.75–1.9 cm. With previous results of righting in the 0-DAH nymphs (height loss of ∼0.3 m; Zeng et al., 2017), we estimated that post-righting, pre-glide downward acceleration took place during a vertical fall of 0.4–1.6 m. Older nymphs exhibited temporal delay and greater downward displacement for these key events (see also below).

### Kinematics of glide initiation

**Fig. 7.**
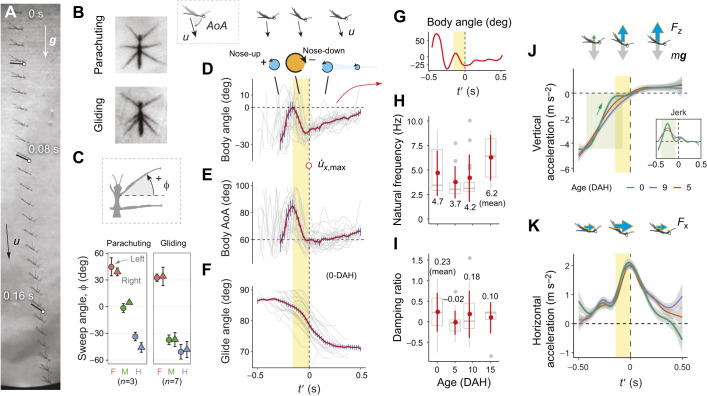
**Kinematics and dynamics of glide initiation.** (A) A representative sequence showing rapid nose-down pitching throughout glide initiation. (B) During glide initiation, the insect swept both mid- and hind-legs posteriorly, as revealed by high-speed video frames before glide initiation and during stationary gliding. (C) A comparison of vertically projected sweep angle (φ; means±s.d.) of six legs during parachuting and gliding. A positive φ corresponds to a forward sweep with respect to transversal. F, M, H represent fore-, mid- and hind-legs, respectively. (D) Body posture angle (with respect to horizontal), (E) body angle of attack (AoA) and (F) glide angle through time relative to maximum horizonal acceleration (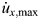), denoted as *t*′. Over a transient period of ∼0.16 s before reaching 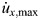 (shaded in yellow), the insect performed nose-down pitching, with body AoA reducing from ∼85 to ∼60 deg, followed by relatively slower upwards pitching and continuous reduction of glide angle. Gray lines are from individual trials; red lines are mean values with s.e.m. (G) Throughout glide initiation (−0.5 s<*t*′<0.5 s), the insect body underwent damped oscillation, here shown with one trial. (H,I) Summary of the natural frequency and damping ratio, respectively. Red dots show means±s.d. (J) Vertical deceleration suggests increased vertical force from −0.4 to −0.1 s (green shading), corresponding with peak jerk at *t*′=−0.28 s (inset), followed by substantial horizontal acceleration from −0.2 to 0.25 s (K). Values represent means, with shadings corresponding to 1 s.e.m.

We now summarize body kinematics during glide initiation, focusing on the 0-DAH nymphs. As shown by a sequence in lateral view (Fig. 7A), the transition from parachuting to gliding was mediated with a nose-down pitching maneuver. High-speed videos in top view showed that posteriorly swept midlegs and hindlegs underwent the most noticeable postural change following glide initiation, as compared with a more laterally oriented posture for both leg pairs during parachuting (Fig. 7B,C; Movie 3).

**Fig. 8.**
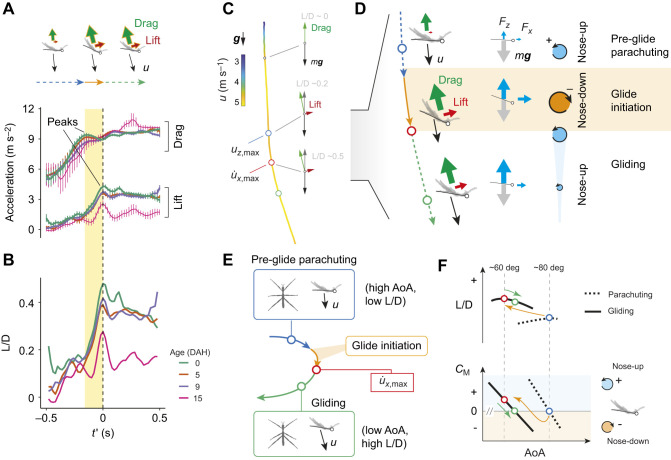
**General mechanism of glide initiation.** (A) Lift and drag peaks are correlated with vertical and horizontal force peaks in Fig. 7. (B) Mean lift-to-drag ratio (L/D) shows a large increase corresponds with horizontal acceleration (Fig. 7K) during glide initiation. (C) A sample trajectory, with color coding representing speed of the center of mass (CoM) and annotated with lift and drag vectors in actual relative proportions. The curved section of the trajectory corresponds to glide initiation, as demonstrated by a schematic (D) showing the transition in body posture (indicated by line segments), CoM velocity and forces. The transitional sequence is characterized as: (1) pre-glide parachuting (blue dashed line), during which terminal speed is reached (blue circle); (2) glide initiation phase (orange line), which ends with peak horizontal acceleration (red circle); and (3) gliding phase (green dashed line and circle). (E) Glide initiation is a controlled transition from one statically stable state to another with reduced angle of attack (AoA) and increased L/D. (F) As depicted by the sequence of blue–red–green circles (i.e. from the orange to the green arrow), such a transition involves an initial reduction and subsequent increase of AoA. During glide initiation, the body first undergoes nose-down pitching (orange shade) and subsequent damping during nose-up pitching (blue shade). *C*_M_, pitching moment coefficient.

Body kinematics during glide initiation are described based on data from the youngest 0-DAH group given their highest agility among all age groups. By aligning body pitch angle (β) with respect to the time of glide initiation (i.e. *t*′=0 s when reaching the maximum horizonal acceleration, 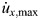), we found a general trend for rapid reduction in β from 0.2±4.3 deg at *t*′=−0.16 s to −19.2±1.2 deg at *t*′=0 s, with a mean angular speed of 118.8 deg s^−1^ (Fig. 7D). This nose-down pitching phase was designated ‘glide initiation’. Temporally coupled with this nose-down pitching was a reduction in body angle of attack (AoA) from ∼80 to ∼60 deg (84.9±4.6 deg at *t*′=−0.16 s; 59.6±1.3 deg at *t*′=0 s; see Fig. S4) at an angular speed of 158.1 deg s^−1^, and a reduction in glide angle from ∼85 to 79 deg (84.6±0.7 deg at *t*′=−0.16 s; 78.8±0.9 deg at *t*′=0 s) (Fig. 7E,F). After *t*′=0 s, the glide angle continued to decline and reached 70.8±0.7 deg at *t*′=0.5 s.

The nose-down pitching during glide initiation (–0.16 s<*t*′<0 s) was accompanied by both prior and subsequent nose-up pitching with reduced amplitude, which together formed a damped oscillation (Fig. 7G). The mean natural frequency was 4.7±2.3 Hz (mean±s.d.) in the 0-DAH group and ranged from 3.7 to 6.2 Hz among all age groups (Fig. 7H). By sampling peaks, we found the mean damping ratio to be 0.23±0.47 in the 0-DAH group and ranging from −0.02 to 0.23 among all age groups (Fig. 7I), suggesting a pattern of underdamping that led to an equilibrium in pitching moment (see Discussion).

### Dynamics of glide initiation

We now describe the force dynamics in the form of accelerations in 0-DAH nymphs. In the vertical direction, we observed a large change in acceleration centered at *t*′=−0.28 s (Fig. 7J), which corresponded to a rapid increase in vertical force production starting at *t*′=−0.4 s. In addition, we observed a peak in horizontal force across the glide initiation phase (from *t*′=−0.2 to 0.2 s) (Fig. 7K).

Examining the dynamics of lift and drag, we found a high-drag phase that preceded the transition to a high-lift generation phase. Specifically, we found a peak of drag at *t*′=−0.14 s (early in nose-down pitching) and a sustained increase in lift throughout the entire nose-down pitching (Fig. 8A). Across the entire nose-down pitching period (yellow shade in Fig. 8A,B), the overall lift-to-drag ratio (L/D) exhibited a rapid increase from 0.16 to 0.48, reaching a peak at the same time as maximum horizontal acceleration (Fig. 8B).

Next, we tested the temporal correlation between body posture, body–flow configuration and glide performance throughout glide initiation. We analyzed cross-correlations between body pitching acceleration (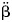), body angle of attack (AoA) and glide angle (θ). We found that the continuous reductions in θ and the increase in β are correlated with negative cross-correlation coefficients (0-DAH, −0.75±0.04; 5-DAH, −0.81±0.03; 9-DAH, −0.71±0.05; 15-DAH, −0.49±0.1; mean±s.e.m.; phase lags <0.05 s) (Fig. S4). Also, the temporal correlation between 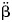 and AoA is indicated by positive cross-correlation coefficients (0-DAH, 0.25±0.03; 5-DAH, 0.20±0.02; 9-DAH, 0.25±0.04; 15-DAH, 0.28±0.05; mean±s.e.m.; phase lag ∼-0.1 s) (Fig. S4).

### Mechanism of glide initiation

Integrating these observations during glide initiation, we characterized the transition across three phases (Fig. 8C,D): (1) pre-glide parachuting, during which the terminal speed is reached and AoA is high (>60 deg); (2) glide initiation phase, as characterized by nose-down pitching and ending with peak horizontal acceleration during nose-up pitching; and (3) gliding phase. In sum, glide initiation is a controlled shift from a low-lift, high-AoA configuration (pre-glide parachuting) to one with high-lift and low-AoA (gliding) (Fig. 8E).

But what possibly underlies the nose-down pitching and damped oscillation throughout glide initiation? For a qualitative assessment, we visualized such a process using schematics. First, we designated two postures: a parachuting posture with high AoA and low L/D (blue circles in Fig. 8F) and a gliding posture with lower trimmed AoA (i.e. when total aerodynamic moment is balanced at the CoM) but greater L/D (green circles in Fig. 8F). As both parachuting and gliding are statically stable, the pitching moment coefficients (*C*_M_) have negative slopes with respect to AoA (lower panel in Fig. 8F).

A rapid switch from the parachuting to the gliding posture (i.e. from the dotted to solid line in Fig. 8F) would create an unstable configuration that undergoes nose-down pitching and subsequent damping (orange arrows in Fig. 8F). In particular, the insect's L/D reached a peak at *t*′=0 s (red circles in Fig. 8F) with an AoA of ∼60 deg (see Fig. 7E). However, because the insect is not trimmed at this AoA (*C*_M_>0), a nose-up pitching moment is generated, which powers the damped oscillation toward an equilibrium (green arrow in Fig. 8F). Such a damped oscillation in pitch is also seen in gliding vehicles switching between statically stable states with different AoA (see Discussion).

### Reduction of gliding performance in first instar nymphs

Reduction in glide capability was evident from the downward shift of kinematic landmarks and the reduction in glide angle (Figs 3, 6). Based on the dynamics of body translation, an increase in terminal speed and a delay of glide initiation increased overall height loss (Fig. 4). For example, comparing the 0-DAH and 9-DAH groups, the mean time to reach terminal speed shifted from 0.26 to 0.55 s, and the mean height shifted from −48 to −83 cm (Fig. 6D,E).

Within different aged first instars, we observed a delay in glide initiation (from 0.55 to 0.8 s) and also a reduction in magnitude of the peak horizontal acceleration (from 2 to 1 m s^−2^) (Fig. 4D). The youngest nymphs also exhibited the strongest vertical acceleration prior to glide initiation (Fig. 7J) and correspondingly the highest peak in lift-to-drag ratio (Fig. 8B). In the horizontal direction, however, the time scale and magnitude of changes in force were similar among age groups. Thus, along with delay in glide initiation, an increased body mass (with greater inertia) also likely weakens vertical accelerations (see Discussion).

### Spatial efficiency of gliding following free falls

**Fig. 9.**
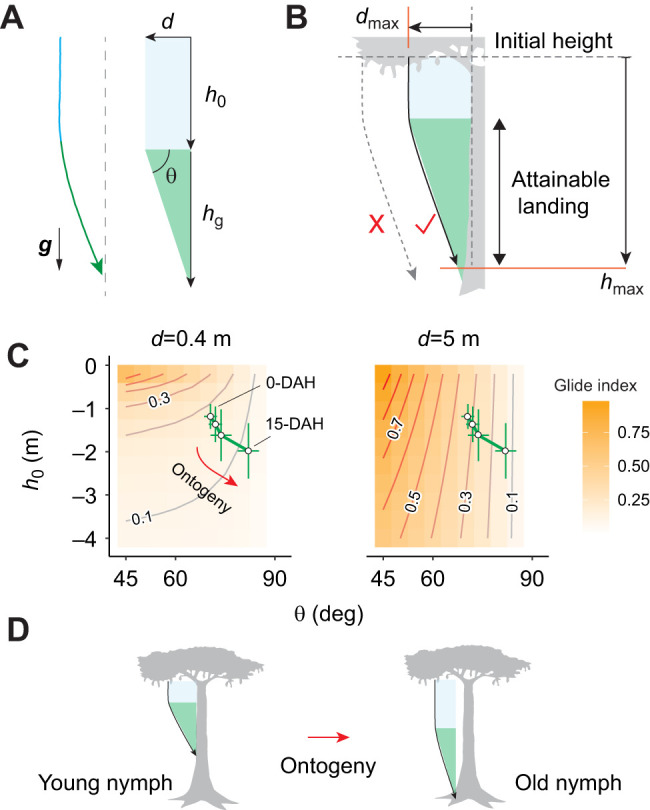
**Spatial efficiency of gliding following free fall.** Spatial efficiency of gliding (i.e. average horizontal travel per unit descent, or the glide index) is determined by four variables: (1) horizontal distance to target tree trunk (*d*); (2) pre-glide height loss (*h*_0_); (3) height loss while gliding (*h*_g_); and (4) height of the under-canopy space (*h*_max_). (A) A simplified configuration of gliding assuming a J-shaped trajectory. (B) Subcanopy space constraints gliding performance, where the maximum of *d* depends on *h*_max_: *d*_max_=(*h*_max_–*h*_0_)tan(θ). (C) Glide index (orange shading) visualized as a function of glide angle (θ) and pre-glide height loss (*h*_0_) for two different horizontal distances (*d*), showing higher variance with greater *d*. Ontogenetic reduction in gliding in the 1st instar nymphs, as represented by shifts in *h*_0_ (Fig. 6E) and in θ (Fig. 3E), is overlaid, with values indicating means±s.d. (D) Schematic demonstration for the ontogenetic reduction of glide performance.

The spatiotemporal pattern of glide initiation, along with ontogenetic variation in glide trajectories, ultimately determine the utility of gliding and the ability to overcome gaps while minimizing height loss. Here, we evaluate determinants of the glide index (i.e. horizontal travel per unit descent) and the context-dependence of the increase in glide angle. Considering a simplified two-dimensional, J-shaped trajectory, and omitting the smooth bending of glide initiation, the glide index can be modeled as a function of three variables: (1) the horizontal distance to target tree trunk (*d*); (2) the pre-glide height loss (*h*_0_); and (3) the height loss in gliding (*h*_g_) (Fig. 9A):
(3)

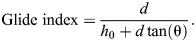


In reality, gliding cannot occur if under-canopy space is of insufficient height (Fig. 9B). Thus, height of the under-canopy space (*h*_max_) constrains the maximum horizontal distance to a potential landing target below *d*_max_=(*h*_max_–*h*_0_)tan(θ).

Within this framework, glide index is thus a function of both the insect's aerodynamic capacity and the location of initial free fall. Given an equivalent *d*, the same glide index can be achieved with different combinations of *h*_0_ and θ. We visualized such variation in glide index as a landscape, which shows that its improvement via reducing glide angle is more effective with greater *d* (Fig. 9C,D). Correspondingly, ontogenetic reduction of glide index in first instar nymphs is more substantial when falling farther horizontally from a tree trunk.

**Fig. 10.**
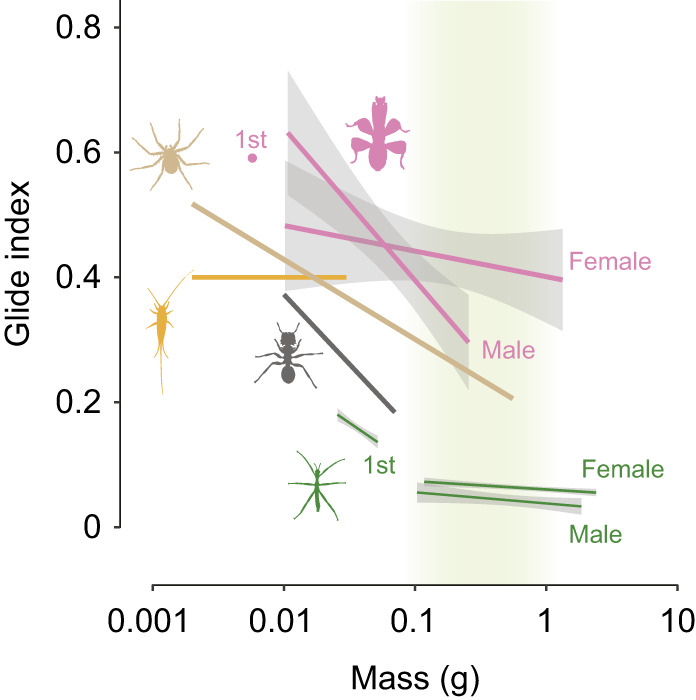
**Variation of glide capability in wingless gliding arthropods.** Glide index as a function of body mass for five well-studied gliding arthropod taxa (see also Table 1). Trend lines are based on least-square linear regressions fitted to the raw data; shading represents 1 s.e.m. (if available). The shaded section (0.1–1 g) corresponds to a reduction in gliding capability unless wing-like extensions are present, as in orchid mantis nymphs (see also Discussion). Note that data from all taxa cover various nymphal stages, except for the gliding ants.

We also sampled gliding capacity in the later-instar nymphs of *E. tiaratum*. They exhibited drastic ontogenetic reduction in gliding capability across a wide range of body masses (0.025–3 g). Their glide index was <0.1 and also declined with increasing mass and wing loading (see Supplementary Materials and Methods, section C). As shown in Fig. 10 and Table 1, compared with all other wingless gliding arthropods studied to date, *E. tiaratum* nymphs exhibited a lower glide index, mainly owing to their slender body–leg sections, high wing loading and the relatively low horizontal distance *d* used in experiments.

**Table 1.**
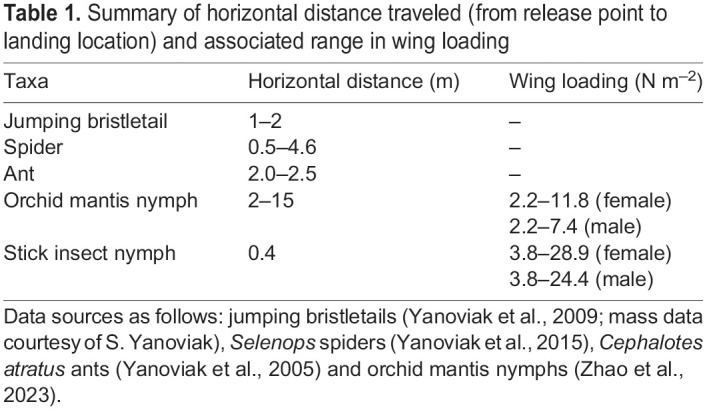
Summary of horizontal distance traveled (from release point to landing location) and associated range in wing loading

## DISCUSSION

### Dynamics of J-shaped trajectories

Following a predominately passive initial falling, the insects quickly began to regulate aerodynamic forces and to use them to perform a series of maneuvers and subsequent gliding. From righting and steering to stable gliding, all midair maneuvers depend on sensory assessment of a number of different environmental cues. Besides gravitational cues and relative air flow used for aerial righting reflexes (Zeng et al., 2017), and visual cues for steering and directed gliding (Yanoviak et al., 2005; Zeng et al., 2015; Socha et al., 2015), our results suggest that sensing of near-zero vertical acceleration may be used as a reference for glide initiation (Fig. 6). The mechanism of this acceleration sensing remains unclear but may be derived from relative speed of airflow or cuticle strain sensed by sensilla on body (Hustert and Klug, 2009; Schmitz et al., 1991), or even optic flow perceived by compound eyes. In addition, integration of various cues may be constantly used in stabilizing body posture; our videos in top view revealed instantaneous stroke movements by legs during maneuvering and gliding (Movie 3).

Our study did not show evidence for landing maneuvers prior to impact (e.g. the swoop-up phase as in gliding lizards; Khandelwal and Hedrick, 2020) in *E. tiaratum* nymphs (Movie 2). This result may partly be due to the limits of visual acuity in small arthropods when approaching relatively large visual targets (at least within our experimental setting) (Caves et al., 2018). Just as flying insects approaching a landing surface (Goyal et al., 2023), a wingless gliding arthropod perceives an optic expansion (i.e. looming) of the landing surface, but may not respond with effective deceleration. Nevertheless, this question can be addressed by future studies with a larger experimental volume allowing for the full development of natural glide angles when falling from trees, along with testing of different target sizes.

Our analyses based on lateral path projection did not assess the dynamics of maneuvering, and our use of kinematic landmarks to partition trajectories can only roughly characterize the rich dynamics of J-shaped trajectories. Future work needs to combine three-dimensional body and leg kinematics to detail underlying behavioral control and associated aerodynamic mechanisms. To model the three-dimensional dynamics of J-shaped trajectories, we should also incorporate variation in initial falling conditions (e.g. body orientation).

### Pre-glide descent in gliding animals

Free fall initiated with either perturbed or self-initiated dropping is likely common in numerous arboreal arthropods, including many non-gliding taxa (Humphreys and Ruxton, 2019; Kane et al., 2021). However, we still lack a general understanding of how these falling movements vary with both body shape and the motions of different body segments and legs among diverse wingless arthropods.

In *E. tiaratum* nymphs, we demonstrated that variable initial body orientation leads to variable heights at which gliding starts (Fig. 6E); such variation is expected to be greater under natural conditions given visual signal noise and wind perturbation. So far, the only comparable data on the spatiotemporal scale of the pre-glide descent are from gliding ants (*Cephalotes atratus*) over a similar mass range (10–80 mg), but these ants required a ∼0.5 s descent over ∼3 m before the glide was initiated (Munk et al., 2015). Laboratory experiments with a vertical wind tunnel revealed that arboreal salamanders need ∼1 s of free fall to reach a terminal speed of ∼10 m s^−1^, with an effective height loss of ∼5 m (Brown et al., 2022). To more fully address the behavioral pathways resulting in different landing heights, future work should record high-resolution three-dimensional body kinematics and consider effect of variation in falling speed, orientation of the body axis and yaw maneuvers.

For gliding animals that need to climb back to the original take-off height, a shorter pre-glide descent and the ability to attain terminal speed as quickly as possible would be favored by selection. Comparative studies are now needed to clarify those behavioral and aerodynamic mechanisms governing the dynamics of this transient phase (see below for the allometry of pre-glide descent). Gliding vertebrates and human wingsuit gliders can achieve very shallow glides, but they rely on take-off jumping to minimize the initial pre-glide loss of height (see Socha et al., 2015; Stöckl et al., 2020). In this regard, it would also be important to examine glide initiation from free falls for different gliding animals, so to better understand the contributions of take-off jumps.

### Aerodynamics of gliding in wingless arthropods

In *E. tiaratum* nymphs, glide initiation involves a rapid, controlled switch between two statically stable states (Fig. 8). The damped oscillation toward an equilibrium in pitching is characterized by temporally coupled shifts in body–flow configuration, an increase in lift-to-drag ratio, and reduction in glide angle. Among gliding vehicles, the closest analogy is probably the multi-modal parafoils that can glide at different descent angles and variable values of AoA. After switching from one statically stable state to another, these parafoils experience damped oscillations (Slegers et al., 2010; Ward et al., 2013). Nevertheless, we expect the dynamics of glide initiation in *E. tiaratum* to be more complex given their small size (and associated aerodynamics), instantaneous sensory feedback and rapid adjustments of leg posture.

The regulation of aerodynamic forces and moments using slender legs in *E. tiaratum* nymphs is quite different from those used by vertebrate gliders with flexible webbing or membranes (e.g. flying squirrels and flying lizards; Bishop, 2006; Khandelwal and Hedrick, 2022). Also, the cuticle-based body–leg systems of arthropods are relatively more rigid than wings, membranes or webbing of gliding vertebrates – this difference may allow the latter to more efficiently overcome perturbations by absorbing and dissipating energy of fluctuating airflows around them.

Future work should address the whole-insect aerodynamic properties at constant body postures, followed by analysis of transitions between states. In particular, given the ubiquity of falls (and jumps) from tree canopies, being able to transition between parachuting and gliding might be essential for wingless arthropods to initiate glides, and also to subsequently maneuver through the sub-canopy space.

### Ontogeny of gliding in wingless arthropods

We show that gliding performance in *E. tiaratum* declined across ontogeny. Across adults and juveniles of gliding taxa, similar negative allometry of gliding capability was reported in both gliding vertebrates and arthropods (e.g. gliding lizards, gliding ants, gliding spiders and orchid mantis; McGuire and Dudley, 2005; Yanoviak et al., 2008; Yanoviak et al., 2015; Zhao et al., 2023).

As shown in the first instar nymphs, the downward shift in numerous kinematic landmarks was associated with an increase in vertical speed (Fig. 4A). Notably, nymphs of different ages exhibited a relative consistent starting time of glide initiation (Fig. 4D), suggesting that there is no ontogenetic reduction in sensory response. In addition, greater moment of inertia in pitch likely reduced the effects of applied aerodynamic torque during maneuvering and glide initiation. Simply put, increased falling speed stretched the J-shaped trajectories vertically, and increased mass reduced body agility required for maneuvering and gliding initiation.

Increased height loss by later nymphs may constrain the utility of gliding, especially if there is limited sub-canopy space. The loss of aerial initiation phase (e.g. self-dropping and jumping behaviors) after 5-DAH may partially compensate for this effect (Zeng et al., 2020). Gliding-related morphology and behavioral mechanisms for the aerial initiation phase are likely to vary both ontogenetically and among species, and will alter gliding performance accordingly. Across all sampled nymphs, we saw a drastic increase in Reynolds number with size (Fig. S6D). Lift generation typically increases with Reynolds number in the range relevant to insect flight (Ellington, 1991), and thus older nymphs may achieve better equilibrium glide angles if sufficient space is available to glide.

### Implications for flight evolution

Our work suggests that falling and gliding may foster selection on traits that enhance aerodynamic force production by wingless arthropods. These traits include both behavioral control of body orientation and the size of aerodynamic structures, such as winglets or flat extensions on the body or legs.

Considering a typical J-shaped gliding trajectory, selection for slower descent and landing at higher points can promote traits that reduce wing loading and enhance maneuverability (i.e. faster righting and steering). On the one hand, quicker righting will ensue from faster body and appendicular movements and mobility (e.g. ranges of motion) that yield useful aerodynamic torque to reorient the body (see Zeng et al., 2017). On the other hand, better steering during descent presumably depends on varying leg postures. The role of pre-glide descent in flight evolution is less obvious than the benefits of equilibrium gliding. Assuming quasi-equilibrium parachuting with vertical force balance *m**g***=0.5ρ*u*^2^*C*_D_*A*, it is trivial to derive 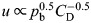. Both a reduction in wing loading and increased body drag will then reduce terminal speed and thus height loss. Thus, aerodynamic surfaces that increase both projected body area and drag coefficients would be beneficial. Notably, differences in drag coefficient may explain why gliding ants (Munk et al., 2015) have a lower wing loading but greater loss in pre-glide height relative to newly hatched *E. tiaratum*.

In the evolutionary transition from apterygotes to pterygotes, a potential role for emergent winglets for reducing pre-glide descent height should be evaluated. Early apterygotes lacked long legs and may have relied more on abdominal motions and their long caudal appendages for postural control (Wootton and Ellington, 1991). Nevertheless, it would be informative to study extant insects with variably sized wings (e.g. different species of adult phasmids; Zeng et al., 2023) to determine how they interact with accelerating flow during free fall, and how pre-glide height loss may vary with wing size. In addition to maintaining statically stable parachuting and gliding, the ability to quickly transition from one to the other likely was critical to early flight evolution.

Given the same pre-glide height loss, a shallower glide angle not only conserves height loss but also allows the animal to take off farther from tree trunks (Fig. 9). If body shape is conserved, reduced gliding capability is the penalty for increased size, which in turn can promote the gain of wings either across ontogeny or in evolutionary time. Both outcomes are relevant to the initial gain of insect wings, given that the earliest winged insects were likely larger (1–2 cm) than their wingless terrestrial ancestors and were characterized by partially winged nymphal stages (Dudley, 2002; Haug et al., 2016; Prokop et al., 2023).

On the ontogenetic scale, an increase of wing size should be favored for better aerial travel. Among wingless gliding arthropods, reduction of gliding capability over a body mass range of 0.1–1 g (Fig. 10, Table 1) can be attributed to the increases in body inertia and falling speeds. It is thus of particular interest for future research to estimate the likely reduction of gliding performance in ancestral apterygotes over this body size range, so as to understand how selection for flight may be reinforced as body size increases. Besides body mass allometry, future studies may evaluate how wings or wing-like structures scale with body size, and can consider body size variation on both ontogenetic and evolutionary scales relative to aerodynamic performance.

## Supplementary Material

10.1242/jexbio.247805_sup1 Supplementary information
