# Supplementary material for: Biomechanics and ontogeny of gliding in wingless stick insect nymphs (Extatosoma tiaratum)
Source: J Exp Biol. 2024 Dec 16;227(24):jeb247805. doi: 10.1242/jeb.247805 (PMC11698037; doi:10.1242/jeb.247805)
Supplement: Supplementary information [file jexbio-227-247805-s1.pdf]

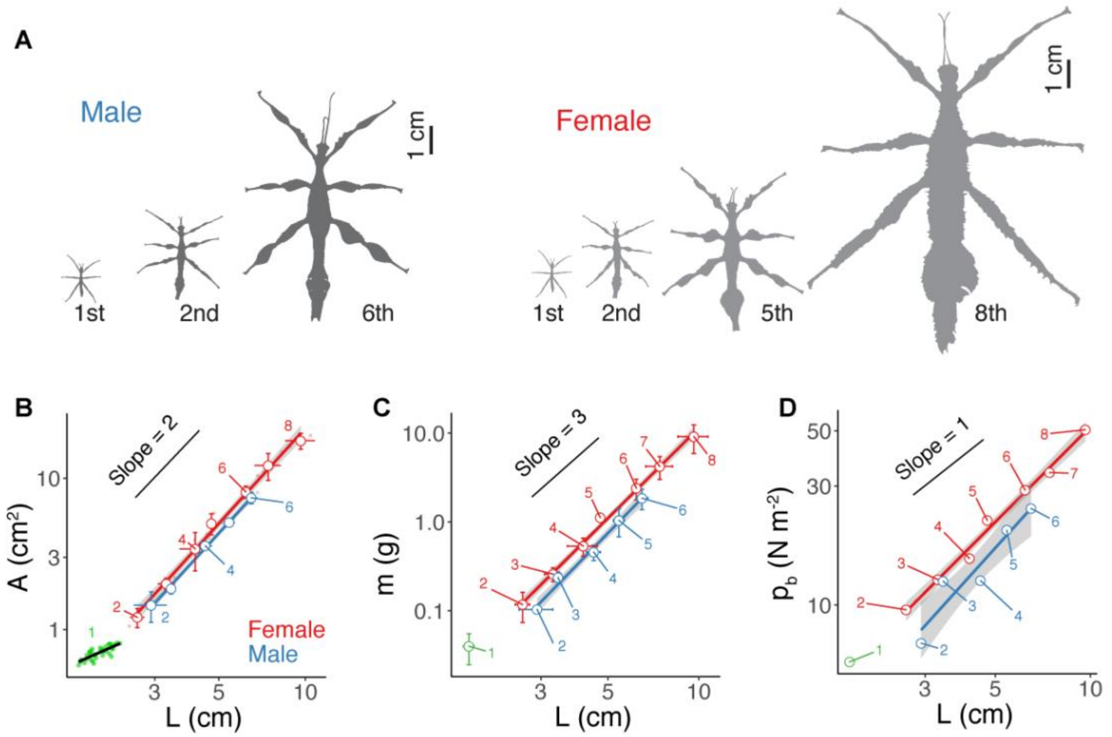

Fig. S1. Ontogenetic variation in gliding-related morphology for *E. tiaratum* nymphs.

(A) Dorsally projected profiles of male and female nymphs at different age, demonstrating a ~100-fold increase in body mass and ~5-fold increase in wing loading. **(B)** Variation of average projected area (A), **(C)** body mass (m), and **(D)** wing loading (p_b) with respect to body length (L). Dots indicates means±S.D; numbers denote instar stages. Trend lines denote linear regression models; colors represent the two sexes. Sex of 1st instars was not ascertainable. Power-law scaling exponents as follows: (1) area: 1st instar, 0.84±0.11 (mean±s.e.m.); later instars, 2.06±0.05 in males, and 2.12±0.06 in females; (2) mass: 3.48±0.20 in males; 3.36±0.10 in females; 3.29±0.16 as sex-averaged; (3) wing loading: 1.40±0.26 in males; 1.27±0.06 in females; 1.27±0.11 as sex-averaged.

Journal of Experimental Biology • Supplementary information

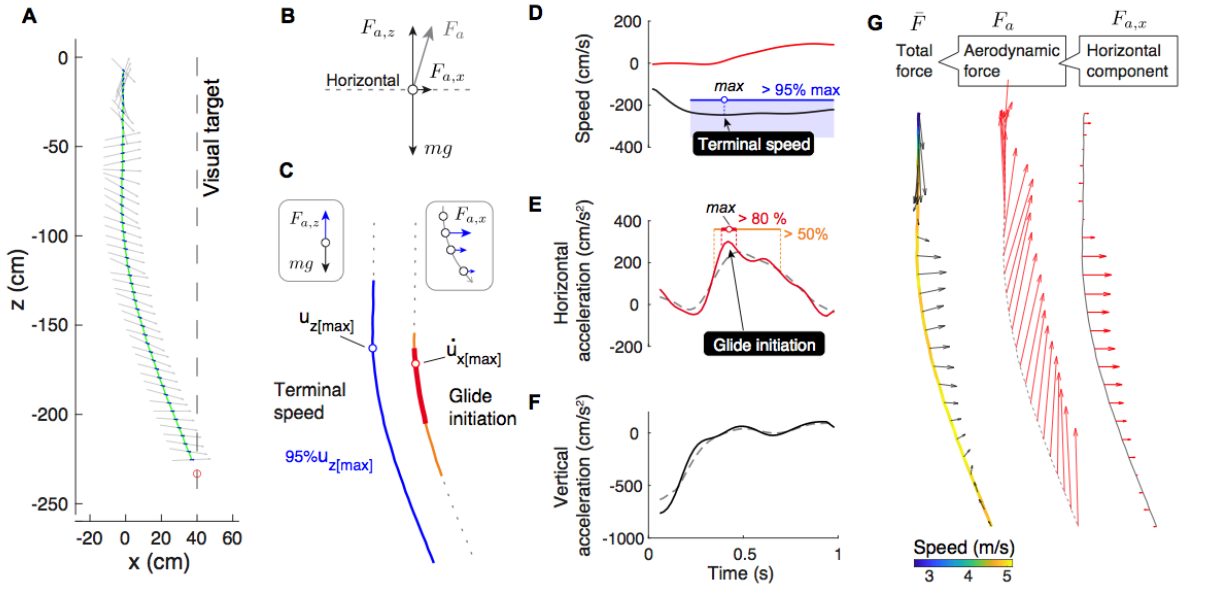

Fig. S2. Phases of gliding along J-shaped trajectories.

(A) Sample trajectory for a 1st instar nymph. The green line represents the approximated center of mass (CM). Gray line segments represent the longitudinal body axis and its orientation, and are elongated for clarity. **(B)** Force diagram in flight. **(C)**. Temporal landmarks in the aerial trajectory: (1) terminal speed (i.e., maximum vertical speed, $u_{z[max]}$), and (2) glide initiation (i.e., the moment of maximum horizonal acceleration, $\dot{u}_{x[max]}$), both as annotated with circles. **(D)** Variation in translational speed through time. The steady-state vertical force balance was identified when $u_z > 95\%\ u_{z[max]}$, indicated as blue regions in both **(C)** and **(D)**. **(E)** Horizontal acceleration ($\dot{u}_x$) with one rapid peak (white circle). The 80% and 50% ranges of horizontal acceleration are indicated in **(C)** and **(E)** in red and orange, respectively. **(F)** Vertical acceleration ($\dot{u}_z$) shows a continued reduction in magnitude as aerodynamic forces increased. In **(E)** and **(F)**, dashed lines indicate a moving-window average (window size, 0.2 s or 10 data points). **(G)** Force vectors mapped on the same trajectory, arranged from left to right as: total force ($\bar{F}$), aerodynamic force (F_a), and the horizontal component of aerodynamic force ($F_{a,x}$).

Journal of Experimental Biology • Supplementary information

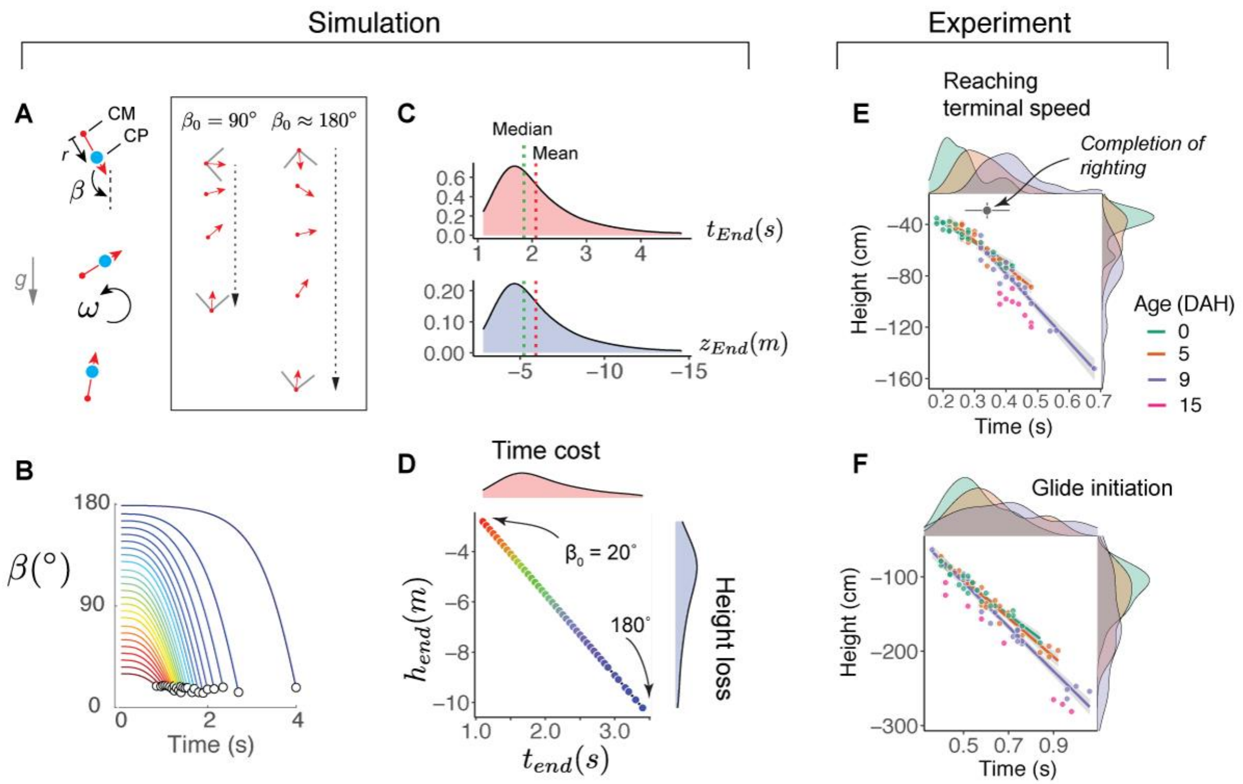

Fig. S3. Pre-glide maneuvers influence when and where gliding is initiated.

We examined the influence of initial body orientation on subsequent aerial behavior using a simplified righting model (see **Appendix B**). The configuration of this simplified two-dimensional model is shown in **(A)**. The righting is generated by the aerodynamic moment via the center of pressure (CP) positioned dorsally to the center of mass (CM) at a distance r. **(B)** shows the dynamics of the body orientation angle (β) with different initial values (β_0, as represented in variable colors). Righting with a greater initial β_0 increases time of righting and yields a greater height loss at the finish **(C)-(D)** Both finishing time (t_{End}) and height loss (h_{End}) are right-skewed (skewness calculated with 'skewness' function from R package 'moments': time, 0.749; height, -0.75;) and are linearly correlated with one another. **(E)-(F)** Time and height loss to reach the terminal speed and at glide initiation as observed in differently aged nymphs; dots represent values from different trials. There were significant linear correlations between these variables for all age groups ($R^2 > 0.80$ for reaching the terminal speed; $R^2 > 0.94$ for glide initiation). The gray dot in **(E)** represent the completion of aerial righting for insects released upside-down under controlled conditions (mean±S.D.; Zeng et al., 2017).

Journal of Experimental Biology • Supplementary information

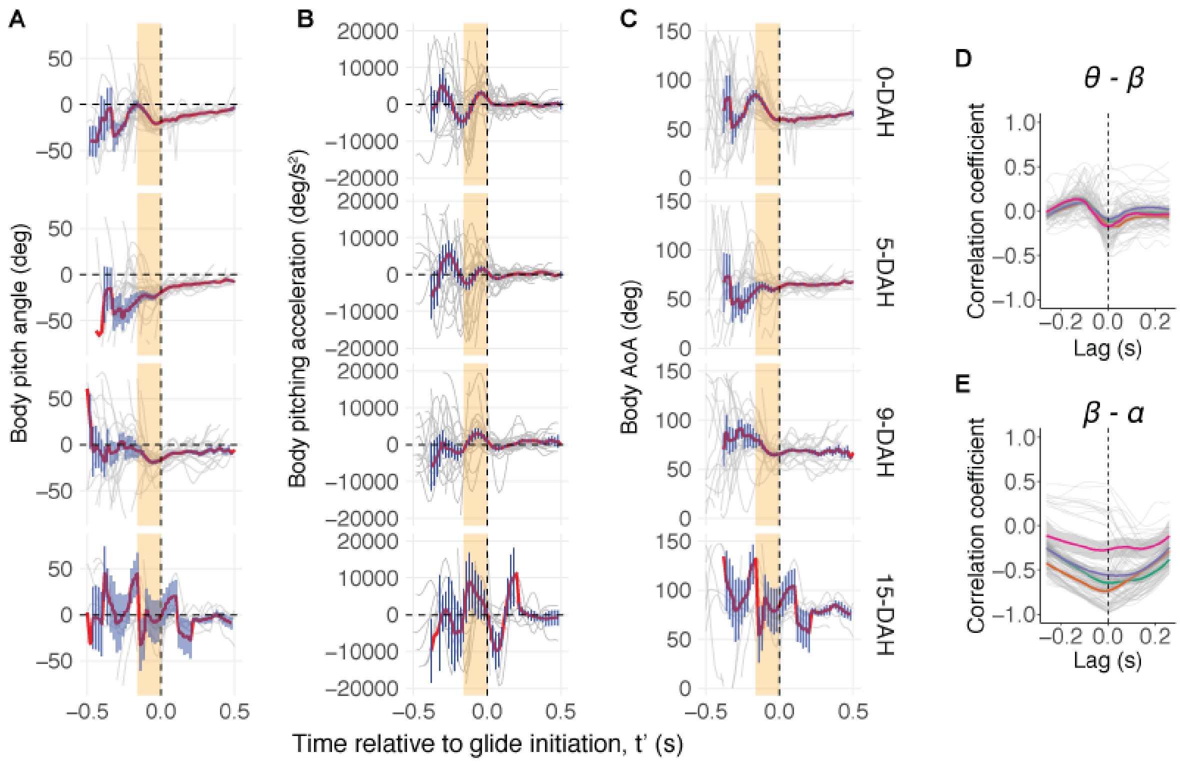

Fig. S4. Body angle relative to horizontal and body angle of attack during glide initiation.

(A)-(C) show variation in body posture angle (β), body pitching acceleration ($\ddot{\beta}$) and body angle of attack (α) throughout glide initiation for different age groups (0-15 DAH), aligned with respect to the peak of horizontal acceleration ($\dot{u}_{x[max]}$). Trend lines are means with error bars representing s.e.m. **(D)-(E)** Temporal correlation of body kinematics: glide angle (θ) vs. body pitch angle (β) and body pitching acceleration ($\ddot{\beta}$) vs. angle of attack (α) over -0.3 s < t' < 0.3 s (see text for details). This figure is supplement to **Fig. 7-8**.

Journal of Experimental Biology • Supplementary information

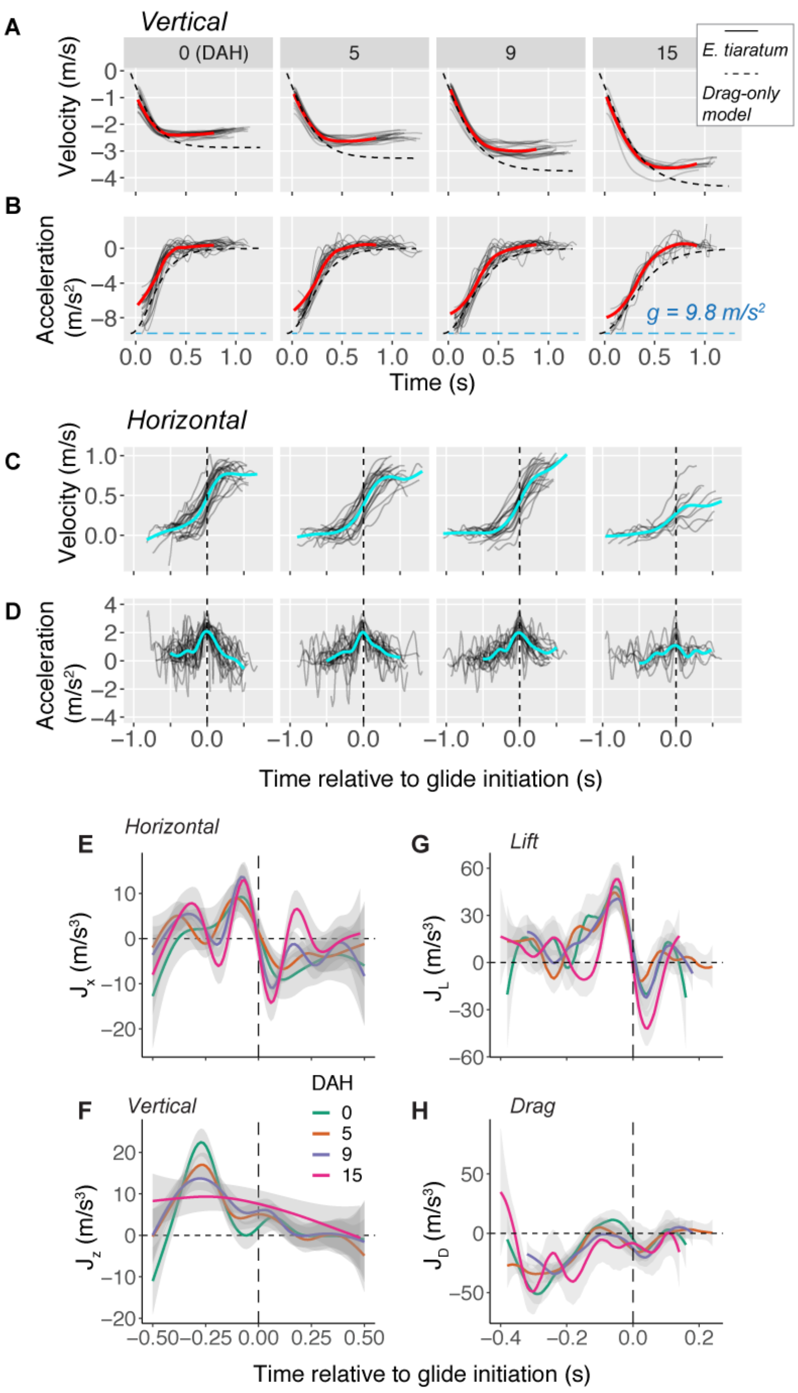

Fig. S5. Velocity, acceleration and jerk along J-shaped trajectories.

(A)-(D) show velocity and acceleration for vertical and horizontal translational components of the center of mass. Colored lines are based on LOESS regression, with data from all trials depicted in the background. These figures are supplements to **Fig. 4A-B** and **Fig. 7J-K**. **(E)-(F)** Horizontal and vertical jerks during glide initiation. Horizontal jerk shows similar timing and magnitude among differently aged insects; vertical jerks show ontogenetic loss in vertical force generation (see **Fig. 7J**). **(G)-(H)** Lift and drag jerks during glide initiation, showing a characteristic lift pulse prior to peak horizontal acceleration (see **Fig. 7K**). Curved are based on LOESS regression, with shading indicating s.e.m.

Journal of Experimental Biology • Supplementary information

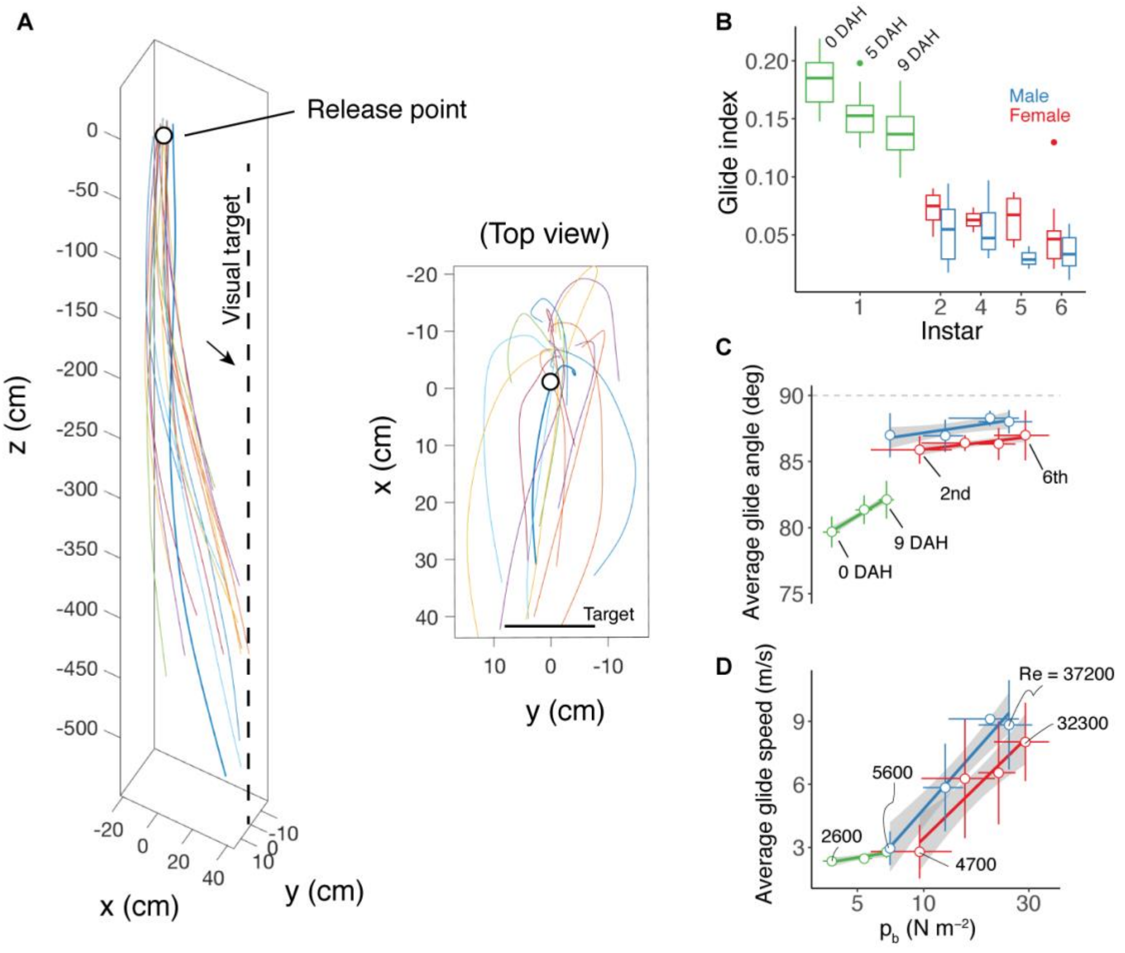

Fig. S6. Ontogenetic reduction in glide performance across instars.

(A) Three-dimensional gliding trajectories of 2nd instar nymphs from oblique and top views. **(B)** Reduction of glide index (i.e., horizontal travel distance per unit descent) across ontogeny. **(C)-(D)** Increase in average glide angle and increase in glide speed with respect to increased wing loading (slopes of regressions: male, 11.8±1.6; female, 10.2±2.1; $P < 0.05$). Reynolds numbers are based on body length.

Journal of Experimental Biology • Supplementary information

Supplementary Materials and Methods

A. Control model - Simulation of falling cylinders

We used a simplified aerodynamic model to compare against the vertical movement of nymphal insects dropped from height (**Fig. 4A**). The model simulates falling of a passive and statically stable cylinder in horizontal orientation, assuming quasi-steady aerodynamics. The cylinder has a ratio of diameter (d) to length (l) of 1:10, and is of the same mass (m) and projected planform area (A) as the 1st instar nymphs (**Fig. S1**). The weight of the cylinder is offset only by aerodynamic drag. The system equations are:

$$\dot{u} = F_D m^{-1} - g$$

$$\dot{z} = u$$

(S1)

where u is falling speed, z is vertical displacement, g is gravitation acceleration, $F_D (= 0.5\rho_{air} u^2 C_D A)$ is aerodynamic drag, and ρ_{air} is air density and C_D is drag coefficient.

We conducted numerical simulations for two models using the Runge-Kutta-Nyström method in MatLab (Mahooti, 2023). The first model incorporates a drag coefficient (C_D) varying with the Reynolds number during descent, following Ellington (1991):

$$C_D = sin(\alpha)^3 \cdot C_n + cos(\alpha)^3 \cdot C_t$$

$$C_n = \left(0.57 + 0.34 \cdot exp\left(-7.6 \cdot \frac{d}{l}\right)\right) \cdot (1.1 + \frac{22}{Re_d})$$

$$C_t = 1.33 \cdot \pi \cdot Re_l^{-0.5} + 2 \cdot \pi \left(\frac{l}{d}\right) \cdot Re_l^{-1}$$

(S2)

where $\alpha (= 90^\circ)$ is angle of attack, Re_d and Re_l are Reynolds number based on diameter and length, respectively; C_n and C_t are force coefficients in the normal and tangential directions, respectively, with respect to the longitudinal axis of the cylinder. As the cylinder is always horizontal (i.e., $\cos(\alpha) = 0$), there is no tangential force.

In the second model, C_D was set as ~0.766, which was derived from the terminal speed of 0-DAH nymphs (2.65 m/s; Re ~1000). With a simulation time span of 1.3 s, both models generated similar displacement profiles with a maximum difference < 5 mm for parameters based on 0-DAH nymphs, and < 2 cm for parameters based on 15-DAH nymphs. Besides providing a reference to show the insects' lift production, this result also suggests that the influence of Reynolds number on drag coefficient can be ignored for of body lengths < 2 cm.

Journal of Experimental Biology • Supplementary information

B. Simulating aerial righting with different initial body orientation

We used a simplified model to demonstrate how variation in initial orientation leads to different completion times and heights while righting. The model is derived from the stereotypical righting posture of nymphal *E. tiaratum*, and consisted of a point mass (CM) with a dorsally positioned center of pressure (CP) (**Fig. S3A**). The body orientation is defined by the angle between the dorsal direction and vertical axis (β). Also, we assumed no influence of Reynolds number on force coefficients and considered only vertical translation. The system equations are:

$$\dot{\omega} = MI^{-1}$$
$$\dot{\beta} = \omega$$
$$\dot{u} = Fm^{-1} - g$$
$$\dot{z} = u$$

(S3)

where $M(= qAC_D r|\sin(\beta_{(t)})|)$ is the aerodynamic moment about CM, $F\ (= qAC_D)$ is the drag force, $q(= 0.5\rho_{air}u^2)$ is dynamic pressure, $\beta_{(t)}$ is body orientation at time t, ω is angular speed, and u is linear speed. The drag coefficient C_D was assumed to be 0.6; other parameters were set the same as a 1st instar *E. tiaratum* (body mass of 2.5 mg, projected area of 65.52 mm^2; moment of inertia of =7.5×10-7 kg/m^2, radius from CM to CP of 4 mm; Zeng et al., 2017). This model demonstrates potential variance on the spatiotemporal scale for the completion of righting, whereas the actual righting involves complex interactions between active leg motions and local air flow.

We performed a numerical simulation using Runge-Kutta-Nyström method in MatLab (Mahooti, 2023), with a range of initial orientations (β_0) between 30˚ – 180˚, and time step of 0.05 s. Righting was assumed to be complete when $\beta \leq 20˚$, which corresponds approximately to the final body orientation in actual insects. We then summarized the distribution of completion time (t_{End}) and height loss (h_{End}) at the conclusion of righting (**Fig. S3A-D**).

Journal of Experimental Biology • Supplementary information

C. Reduction of gliding in later instars

Method Experiments were conducted in the northwestern staircase of the Valley Life Sciences Building at UC-Berkeley. A 15 cm wide and 9 m long black felt stripe was used as the landing target. All insects were dropped at 40 cm horizontally from the landing target. More specifically, 2nd instar nymphs were dropped from Teflon-coated cups and were filmed with two high-speed cameras. Insects were then tracked at the mid-point of body, and three-dimensional trajectory reconstruction was completed with a commercial software (ProAnalyst, Xcitex Inc., MA, USA). For all later instars, given the significant reduction of gliding capability, the experimenter first held the insect at mid-thorax and then dropped the insect in right-side-up orientation with the head orienting toward the landing target. We used one camera in top view to record the time lapse between release and landing. The landing height was recorded by the experimenter after each trial. The average glide angle and average glide speed were calculated based on a vector from the release point to the landing point.

Results Different from the 1st instar nymphs, the later instars showed near-isometric growth in projected planform area and mass (**Fig. S1**). From 2nd to 6th instars, wing loading (p_b) (i.e., the aerodynamic loading on body-leg system) exhibited a sex-averaged exponent of ~1.3, and varied from 5.9 N m^{-2} in the 1st instar nymphs to 24.4 N m^{-2} in the 6th instar males, reaching a value of 50.5 N m^{-2} in 8th instar females (**Fig. S1**).

Later nymphs (2nd, 4th, and 6th) dropped in front of the same visual target were also capable of gliding (**Fig. S6A**). Compared to 1st instar nymphs, glide indices of later instars were lower, and typically less than 0.1 (**Fig. S6B**). Average glide angles were > 85˚, and decreased with increased wing loading (**Fig. S6C**). Also, average glide speed increased from ~3 m/s to ~9 m/s, with a steeper increase in males. For the average gliding speed, the Reynolds number based on body length increased fourteen-fold, from 2600 in 0-DAH 1st instars to ~37000 in 6th instar females (**Fig. S6D**).

Supplemental references

Ellington, C. P. (1991). Aerodynamics and the origin of insect flight. *Adv. Insect Physiol* **23**, 171-210.

Mahooti, **M.** (2023). Runge-Kutta-Nystrom (https://www.mathworks.com/matlabcentral/fileexchange/55616-runge-kutta-nystrom), MATLAB Central File Exchange.

Zeng, Y., Lam, K., Chen, Y., Gong, M., Xu, Z. and Dudley, R. (2017). Biomechanics of aerial righting in wingless nymphal stick insects. *Interface Focus* **7**, 20160075.

Journal of Experimental Biology • Supplementary information

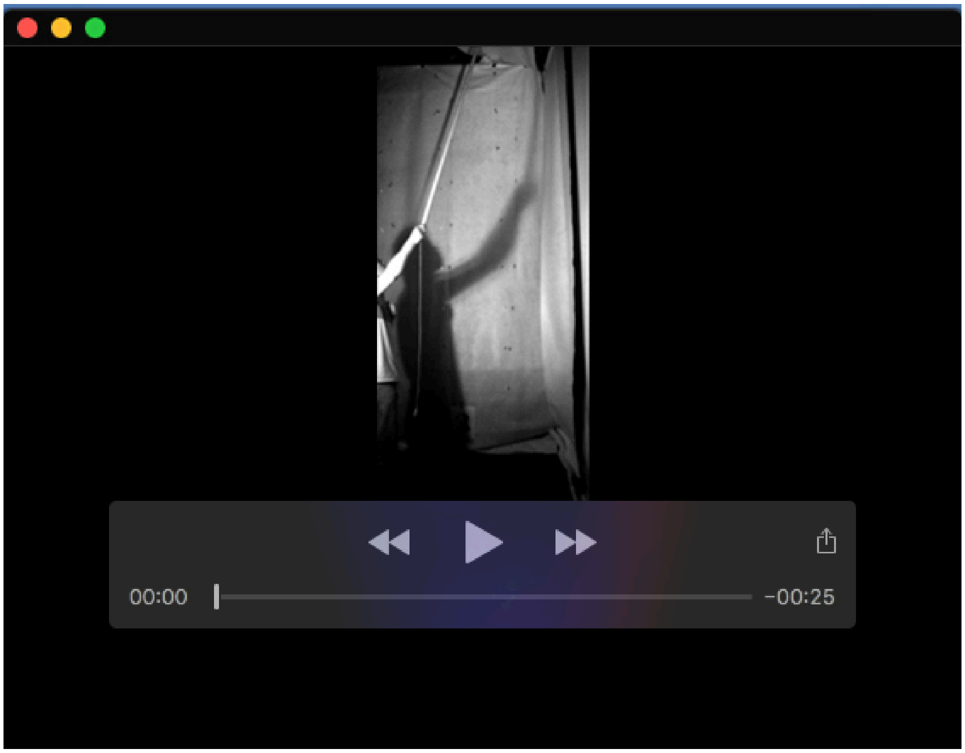

Movie 1. Gliding performance in lateral view.

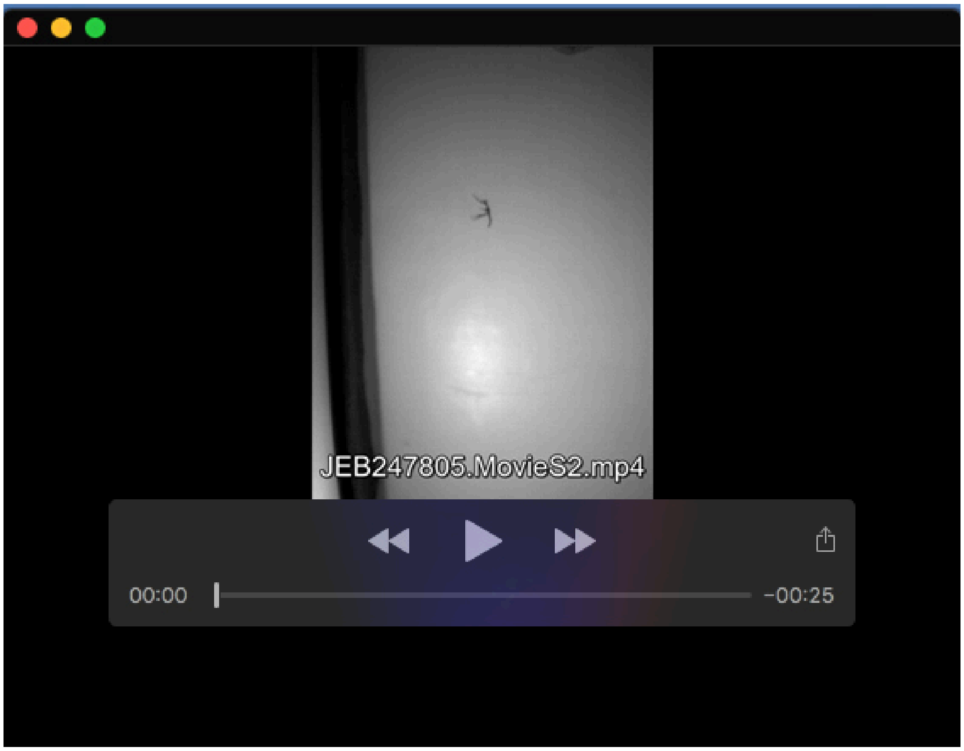

Movie 2. Aerial righting after being dropped from Teflon-coated cups. Close-up of landing in lateral view.

Journal of Experimental Biology • Supplementary information

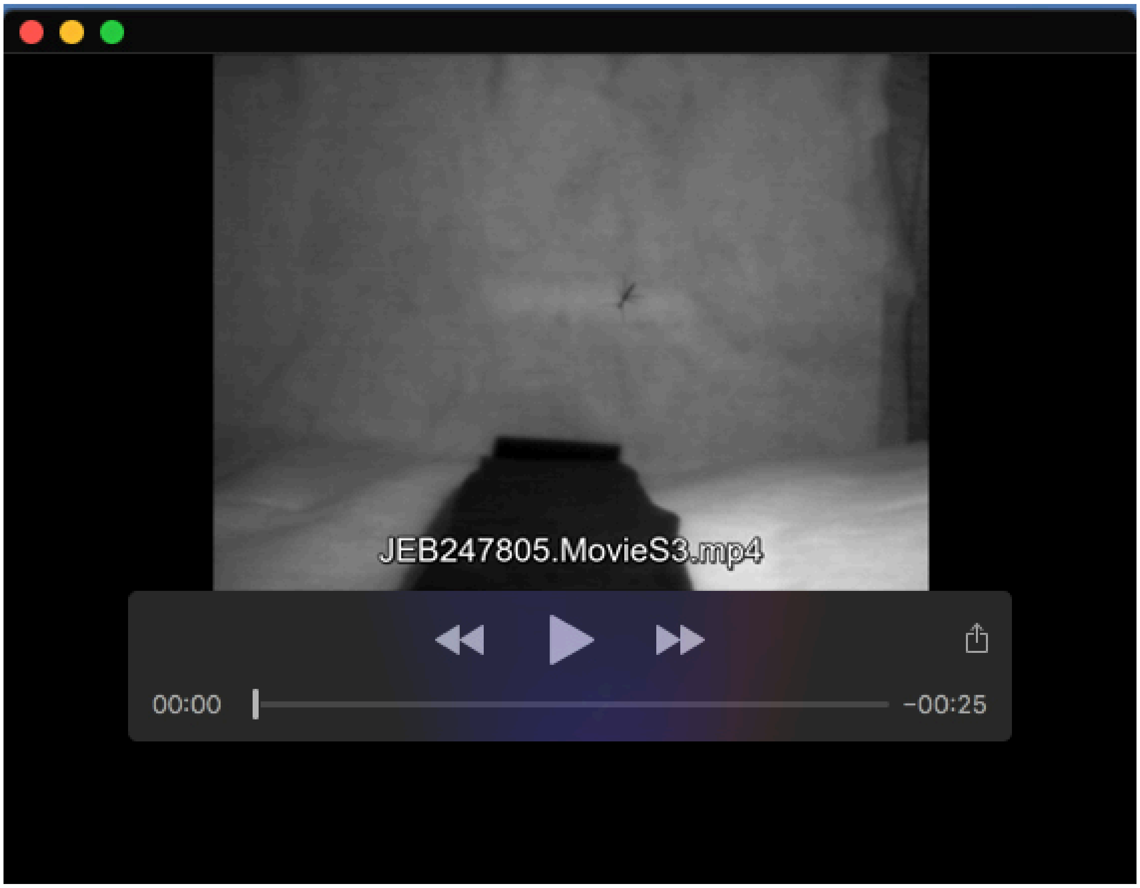

Movie 3. Glide initiation in top view. Midair maneuvering in top view.
